# Development of a counterselectable system for rapid and efficient CRISPR-based genome engineering in *Zymomonas mobilis*

**DOI:** 10.1186/s12934-023-02217-9

**Published:** 2023-10-13

**Authors:** Yanli Zheng, Hongmei Fu, Jue Chen, Jie Li, Yuejie Bian, Ping Hu, Lei Lei, Yihan Liu, Jiangke Yang, Wenfang Peng

**Affiliations:** 1 College of Life Science and Technology, Wuhan Polytechnic University, Wuhan, 430023 P. R. China; 2 Key Laboratory of Industrial Fermentation Microbiology, Ministry of Education, Tianjin Key Laboratory of Industrial Microbiology, The College of Biotechnology, Tianjin University of Science and Technology, Tianjin, 300457 P. R. China; 3 State Key Laboratory of Biocatalysis and Enzyme Engineering, Hubei Engineering Research Center for Bio-enzyme Catalysis, Environmental Microbial Technology Center of Hubei Province, School of Life Sciences, Hubei University, Wuhan, 430062 P.R. China

**Keywords:** *Zymomonas mobilis*, CRISPR-Cas genome engineering, Scarless mutagenesis, clmPheS counterselection marker, Multi-round multiplex genome editing, Native plasmids editing

## Abstract

**Background:**

*Zymomonas mobilis* is an important industrial bacterium ideal for biorefinery and synthetic biology studies. High-throughput CRISPR-based genome editing technologies have been developed to enable targeted engineering of genes and hence metabolic pathways in the model ZM4 strain, expediting the exploitation of this biofuel-producing strain as a cell factory for sustainable chemicals, proteins and biofuels production. As these technologies mainly take plasmid-based strategies, their applications would be impeded due to the fact that curing of the extremely stable plasmids is laborious and inefficient. Whilst counterselection markers have been proven to be efficient for plasmid curing, hitherto only very few counterselection markers have been available for *Z. mobilis*.

**Results:**

We constructed a conditional lethal mutant of the *pheS* gene of *Z. mobilis* ZM4, clmPheS, containing T263A and A318G substitutions and coding for a mutated alpha-subunit of phenylalanyl-tRNA synthetase to allow for the incorporation of a toxic analog of phenylalanine, *p*-chloro-phenylalanine (4-CP), into proteins, and hence leading to inhibition of cell growth. We demonstrated that expression of clmPheS driven by a strong P_*gap*_ promoter from a plasmid could render the *Z. mobilis* ZM4 cells sufficient sensitivity to 4-CP. The clmPheS-expressing cells were assayed to be extremely sensitive to 0.2 mM 4-CP. Subsequently, the clmPheS-assisted counterselection endowed fast curing of genome engineering plasmids immediately after obtaining the desired mutants, shortening the time of every two rounds of multiplex chromosome editing by at least 9 days, and enabled the development of a strategy for scarless modification of the native *Z. mobilis* ZM4 plasmids.

**Conclusions:**

This study developed a strategy, coupling an endogenous CRISPR-based genome editing toolkit with a counterselection marker created here, for rapid and efficient multi-round multiplex editing of the chromosome, as well as scarless modification of the native plasmids, providing an improved genome engineering toolkit for *Z. mobilis* and an important reference to develope similar genetic manipulation systems in other non-model organisms.

**Supplementary Information:**

The online version contains supplementary material available at 10.1186/s12934-023-02217-9.

## Background

*Zymomonas mobilis* possesses several attractive physiological features that make it an ideal platform organism for the production of biofuels and biochemicals from lignocellulosic biomass [1, 2]. For instance, this bacterium is generally regarded as safe (GRAS) and able to accommodate to a wide pH range (pH 3.5–7.5); and as a natural ethanologen having evolved specifically to fit high sugar and alcohol environments, *Z. mobilis* is of great capability in fermenting different biomass sugars for high-concentration ethanol production [3]. More importantly, there is no requirement for controlled aeration during fermentation as *Z. mobilis* is a facultative anaerobe, therein reducing production costs. Other than ethanol, *Z. mobilis* also has endogenous metabolic pathways producing other metabolic byproducts, such as isobutanol, sorbitol, levan, glycerol, as well as gluconic, lactic, succinic, and acetic acids [4].

During the past years, in order to fully unlock the capabilities of *Z. mobilis* in biorefinery to fit the growing global demand for alternative sustainable biofuels and biochemicals, methods have been developed for rapidly and efficiently modifying genes or metabolic pathways to attain industrial applications of this organism. In most of these methods, an antibiotic resistance gene was designed to be flanked by two DNA fragments respectively upstream and downstream of a gene of interest, which, once delivered by a suicide plasmid into the host cells and integrated into their chromosomes via homologous recombination, can replace the target gene and allow the screening of antibiotic-resistant recombinants [5, 6]. It therein leads to the accumulation of antibiotic resistance genes, which, together with the fact that *Z. mobilis* is naturally resistant to several antibiotics [7, 8], may limit multi-round genetic manipulations in this bacterium. Several approaches have been attempted to address the issues associated with using antibiotic resistance genes in *Z. mobilis*, including the use of a GFP fluorescent indicative marker to take the place of using antibiotic resistance genes. However, it would be a time-consuming process since the desired mutants have to be enriched and then identified as non-fluorescent cells by FACS [9, 10]. Other methods also include using the FLP/FRT site-specific recombination system to remove the chromosomally integrated antibiotic resistance cassette while generate gene deletion [11]. An obvious limitation of this method is that an FRT site would be left at the modified locus and possibly interfere with subsequent rounds of genetic modifications in the same host, e.g. recombination events would take place between accumulated FRT sites leading to unwanted chromosomal changes [12].

Recently, a genome editing toolkit based on the endogenous CRISPR-Cas of *Z. mobilis* ZM4 has been established. While it has helped achieve efficient genomic modifications without integration of any antibiotic resistance gene and represents one of currently the most efficient genetic manipulation tools for *Z. mobilis* [13, 14]; it, unfortunately, could not enable successful editing of the native plasmids, because the plasmids were proven to be not essential for cell viability and CRISPR-targeting on them thereby resulted in their loss [15]. Moreover, according to our previous experimental experiences, it is really tedious and time-consuming to cure the stable genome editing plasmids, normally taking about two weeks to get them lost in at least three rounds of inoculating, culturing and PCR-checking. These therefore arise the need of counterselection for clean genetic manipulations in *Z. mobilis*.

Counterselection upon *sacB* has been ever used in *Z. mobilis* to facilitate the loss of integrated plasmid without leaving a scar [16], but unfortunately many *Z. mobilis* strains already harbor a *sacB* gene [17, 18], being probably the reason for the poor positive rate of this approach and thus questioning its general utility. It, and other genes, such as *pyrF* [19] and *glkA* [20], did function in counterselection, but only in the corresponding null mutants, namely requiring pretreatment of the wild-type host cells. Some toxic genes including *mazF* [21] and *hicA* [22], being applied as suicidal markers for counterselection, have been accumulated in the recent literature, which, however, might not be applicable for *Z. mobilis* as thus far in this bacterium an inducible promoter amenable for tightly controlling the toxin expression is still missing.

Besides, in recent years, several studies have reported the use of a PheS variant for successful counterselection in various bacteria in a host-independent manner [23–29]. PheS is the alpha-subunit of phenylalanyl-tRNA synthetase encoded by the *pheS* gene capable of phenylalanine aminoacylation; whilst its derivative, PheS* (PheS with T251A and A294G substitutions), prefers to aminoacylate an analog of phenylalanine, *p*-chloro-phenylalanine (4-CP) in *Escherichia coli* [30, 31]. The lethality of incorporating 4-CP into proteins exhibited robust counterselective pressure [30]. Interestingly, *pheS* genes are present and highly conserved across bacterial species, paving the possibility to develop a PheS variant-based counterselctable marker for *Z. mobilis*. Application of such a counterselection marker has not been attempted in *Z. mobilis* yet. In this regard, here we attempted developing the *pheS* gene of *Z. mobilis* ZM4 (*ZMO1514*) as a counterselection marker upon mutating it to express the conditional-lethal PheS variant, clmPheS. With its assistance, rapid multi-round CRISPR-based engineering of the chromosome, as well as the native plasmids, has been efficiently accomplished in *Z. mobilis* ZM4, providing an improved versatile genetic manipulation toolkit for strain development and gene function demonstration in this bacterium, and also offering an important reference for developing methods for fast scarless genome modifications in other microorganisms.

## Methods

### Strains, growth conditions, and electroporation of *Z. mobilis*

*Z. mobilis* ZM4 and derivatives constructed in this work were listed in Table S1. *Z. mobilis* cells were grown at 30ºC in an RMG medium (20 g/L glucose, 10 g/L yeast extract, 2 g/L KH_2_PO_4_). If required, for *Z. mobilis,* spectinomycin and/or chloramphenicol was supplemented to the final concentrations of 200 and 50 µg/mL, respectively; while for *Escherichia coli*, spectinomycin or ampicillin was supplemented to a final concentration of 50 µg/mL. Competent cells of *Z. mobilis* were prepared as previously described [32] and transformed with plasmids by electroporation using Bio-Rad Gene Pulser (0.1-cm gap cuvettes, 1.6 kV, 200 W, 25 µF) (Bio-Rad, Hercules, CA, USA) following the method developed for *Z. mobilis* [33]. Electroporated cells were incubated in the RMG medium for 3 h at 30ºC prior to plating.

### Construction of plasmids

All the genome editing plasmids, including the chromosome editing palsmids (pCEs) and constructs for the editing of native plasmids (pPEs), were generated to individually include an artificial CRISPR on the pL2R plasmid vector following the previously described method [13]. The *pheS* gene (*ZMO1514*) with mutations, and donor DNA fragments each containing a mutant allele of a target gene, were generated by splicing and overlap extension PCR (SOE-PCR) [34] and individually cloned into the pCE plasmids through the T5 exonuclease-dependent DNA assembly (TEDA) method [35], giving complete pCEs with each carrying a clmPheS counterselection marker. Each of the pPE plasmids includes 4 DNA stretches. For pPE-*dctA* construction, the 4 DNA fragments are the first 327 bp coding sequence of *dctA* (*ZMOp33x010*) as a gene arm (G-arm), the chloramphenicol resistance gene (*cmr*) as a positive-selection marker (M), and two 300-bp sequneces immediately upstream and downstream of *dctA* as left and right arms (L-arm and R-arm), respectively. Of them, the G-arm is 5’-terminally fused to the *cmr* gene forming a fusion marker gene. These DNA fragments was connected by SOE-PCR and used as a donor of the pPE-*dctA* construct for mediating knockout of the *dctA* gene located on the pZM33 native palsmid [36]. The same strategy was also taken for the construction of the pPE-*hsd* plasmid for directing knockout of a 3-gene operon (*ZMOp32x025, ZMOp32x026* and *ZMOp32x028*) encoding a restriction and modification system [6] borne by the pZM32 native plasmid [36].

All plasmids were listed in Table S2. All oligonucleotides were synthesized from GenScript (Nanjing, China) and listed in Table S3. Restriction enzymes and T5 exonuclease were purchased from New England Biolabs (Beijing) Ltd (Beijing, China).

### Construction and screening of mutants, and curing of genome editing plasmids

Genome editing plasmids for chromosomal modifications were individually introduced into *Z. mobilis* cells. Electroporated cells were spread on RMG agar plates containing spectinomycin (RMGSp) at a final concentration of 200 µg/mL and incubated at 30ºC until colonies were seen. Cells of the transformants were grown up in an RMGSp medium, and then spread on an agar plate with 0.2 mM 4-CP (RMGCp) but without spectinomycin. Cells that have formed colonies are regarded as those lost the genome editing plasmid. For native plasmids editing, the pPE constructs were individually transformed into *Z. mobilis* cells via electroporation, and the electroporated cells were spread on RMG agar plates containing spectinomycin and chloramphenicol (RMGSpCm) at final concentrations of 200 and 50 µg/mL, repsectively. Cells of pPE transformants were grown up in an RMGSpCm medium and then spread on an RMGCp agar plate without any antbiotics until colonies were observed. Mutant candidates were screened by colony PCR using primers listed in Table S3. The resulting PCR products were analysed by agarose gel electrophoresis and confirmed by Sanger sequencing (GenScript, Nanjing, China).

### 4-CP sensitivity assay

To assay the sensitivity of *Z. mobilis* strains to 4-CP, growth inhibition test was conducted. Overnight cultures of *Z. mobilis* strains were diluted in fresh RMG medium and grown to OD_600_ of 0.4. Then, each of the culture was serially 10-fold diluted up to 10^− 5^, and 5 µL of each dilution was spotted onto RMGCp agar plates where different concentrations of 4-CP were supplemented. The growth of each strain was photographically recorded after 72-hour incubation at 30ºC.

### FACS analysis

Cells were washed with phosphate buffered saline (PBS) twice, resuspended into PBS to a concentration of 10^7^ cells/mL, and analyzed by flow cytometry using Beckman CytoFLEX FCM (Beckman Coulter, Inc., USA) with the phosphate buffered saline as the sheath fluid. The cells fluorescence of mCherry was excited with the 561 nm and detected with PC5.5 [37].

### Transcriptional analysis of the native plasmids-borne genes in *Z. mobilis*

RNA-seq raw data were processed and quality-controlled using FastQC program; nucleotides with quality scores below 30, as well as adapter sequences, were removed with the Trim Galore tool [38]. Genebank files of the complete sequences of the four native plasmids of *Z. mobilis* ZM4 (CP023716, CP023717, CP023718, and CP023719) were converted into GFF format with the EMBOSS program [39]. Then Hisat2 [40] was employed to build indices that are necessary for mapping the plasmid genes. Gene counting upon annotation information was conducted via the featureCounts tool [41], and gene expression data were calculated as RPKM values, which were subsequently log_2_-transformed and normalized to compare the expression of *ZMOp33x009* gene as well as the mean and median values of overall gene expression. The GEO accession number for transcriptomic analysis in this work that has been deposited into NCBI is GSE242573.

## Results and discussion

### *Z. mobilis* cells that express clmPheS are extremely sensitive to 4-CP

**Fig. 1:**
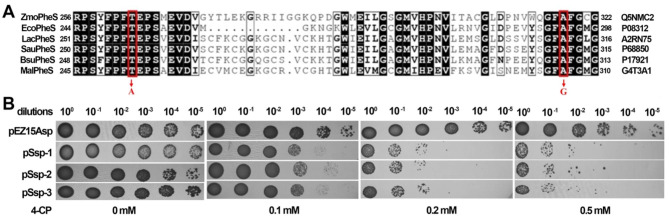
Construction of the clmPheS counterselection marker. (**A**) Multiple sequence alignment of PheS orthologs derived from *Zymobilis mobilis* ZM4 (*Zmo*PheS), *Escherichia coli* K12 (*Eco*PheS), *Lactococcus lactis* MG1363 (*Lac*PheS), *Staphylococcus aureus* MW2 (*Sau*PheS), *Bacillus subtilis* 168 (*Bsu*PheS), and *Methylotuvimicrobium alcaliphilum* 20Z (*Mal*PheS). Red boxes indicate the conserved residuals being subjected to mutagenesis. (**B**) 4-CP sensitivity of *Z. mobilis* transformants. Cell cultures of transformants either harboring the shuttle vector pEZ15Asp, or the clmPheS-expressing plasmid pSsp, were serially 10-fold diluted. Dilutions were spotted onto agar plates containing 4-CP at the indicated concentrations

Based on the annotated genome sequence of *Z. mobilis* ZM4 [36], the wild-type PheS encoding gene *pheS* (*ZMO1514*) was identified. Amino acid sequence alignment of the PheS from *Z. mobilis* ZM4 (*Zmo*PheS) with several PheS proteins of other bacterial species was performed, revealing that the T263 and A318 residues of *Zmo*PheS are the counterparts corresponding to the T251 and A294 residues of the PheS from *E. coli* (*Eco*PheS) [31], respectively (Fig. 1A). Therefore, substitutions of T263A and A318G were made to *Zmo*PheS, generating the clmPheS. Afterwards, a clmPheS expression cassette was cloned onto the *E. coli*-*Z. mobilis* shuttle vector pEZ15Asp [32], yielding the pSsp plasmid, where the strong P_*gap*_ promoter [42] was employed to drive clmPheS expression.

We electroporated pSsp into *Z. mobilis* cells and examined the 4-CP sensitivity conferred by clmPheS to the transformants. Growth inhibition of 3 randomly picked transformants was then assayed with RMGSp plates respectively containing 0, 0.1, 0.2, and 0.5 mM of 4-CP. As shown in Fig. 1B, without 4-CP, transformants of both pSsp and pEZ15Asp (as a reference) were normally and evenly grown up, including the 10^− 5^ dilutions. Strikingly, as the concentration of 4-CP was gradually increasingly supplemented, the growth inhibition effect on the pSsp transformants became clearer. Very few cells from the 10^3^-fold diluted pSsp transformants could grow up when spotted on RMG agar plates containing 0.2 mM of 4-CP (RMGCp), whereas under the same condition the pEZ15Asp-bearing cells kept growing up normally. These results suggested that clmPheS can be employed as an efficient counterselectable marker for *Z. mobilis*, and we therefore used 0.2 mM of 4-CP for counterselection in our subsequent work.

### Plasmid removal is efficiently achievable by using clmPheS for counterselection

**Fig. 2:**
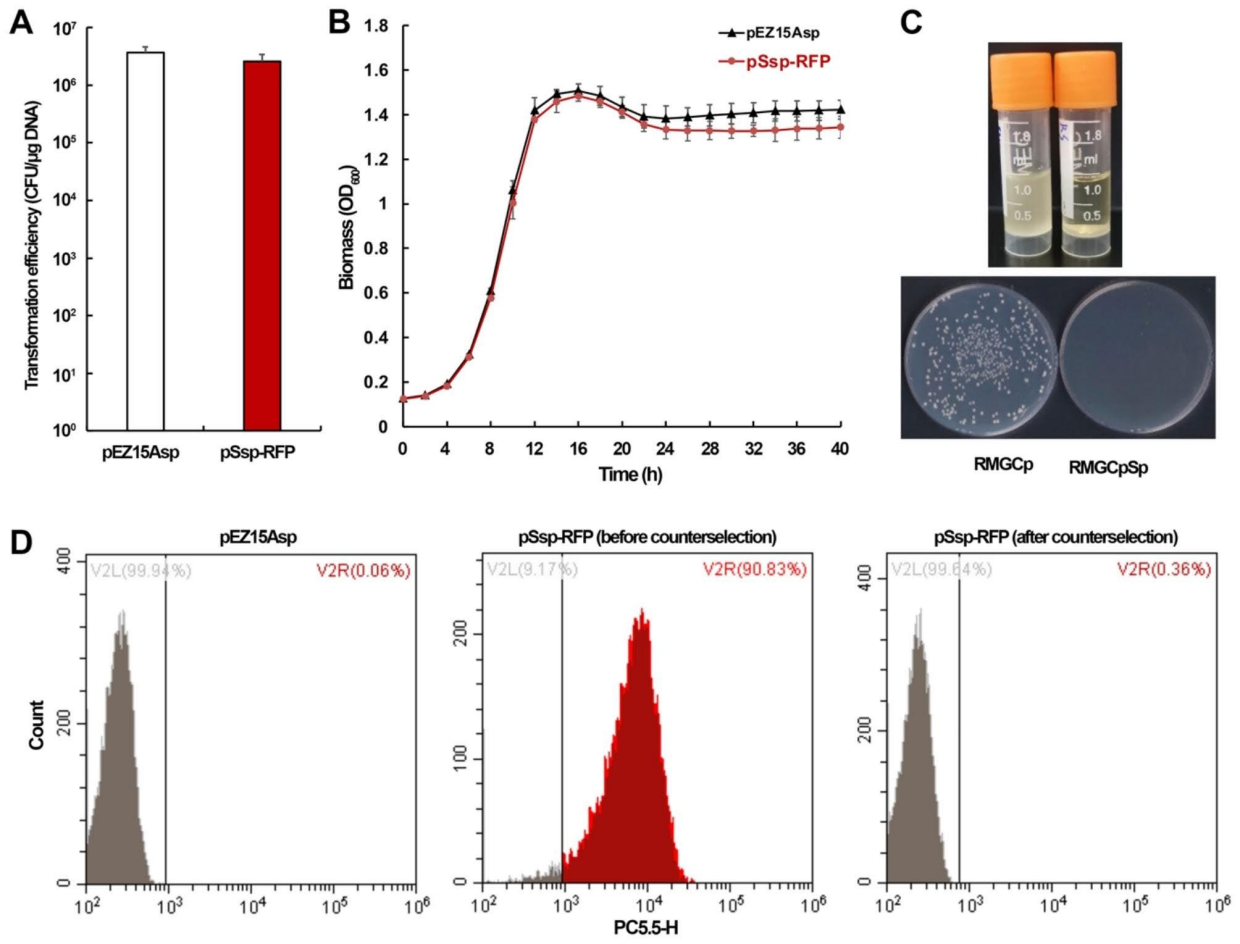
Plasmid curing using the clmPheS as a counterselection. (**A**) Transformation efficiencies of the shuttle vector pEZ15Asp and the clmPheS-expressing plasmid pSsp-RFP. Three replicates were performed for each DNA sample. (**B**) Growth curve measurements of the pEZ15Asp and pSsp-RFP transformants. Three replicates were performed for the experiment. (**C**) Examination of the counterselection effect of 4-CP on plasmid curing from the pSsp-RFP transformant. (**D**) Detection of RFP signal in cells of the pEZ15Asp transformant, and that of the pSsp-RFP transformant before and after 4-CP counterselection

To verify the feasibility of clmPheS for counterselection, we inserted an mCherry red fluorescent protein (RFP) expression cassette in the pSsp plasmid, generating pSsp-RFP, and monitored its presence/absence in cells by measuring the RFP fluorescence intensity through flow cytometry analysis. The pSsp-RFP plasmid, and the pEZ15Asp vector as a reference, were then individually introduced into *Z. mobilis* cells via electroporation, yielding the same level of transformation efficiencies (Fig. 2A). The transformants of both plasmids exhibited comparable growth to each other, indicating that the expression of clmPheS had no obvious negative effect on cell growth when 4-CP were not supplemented (Fig. 2B).

Cells of pSsp-RFP transformants were then randomly chosen and transferred into a liquid RMGCp medium or RMGCp with spectinomycin (RMGCpSp) for 24-hour incubation. As shown in Fig. 2C, they could not grow up in RMGCpSp while grew normally in RMGCp (upper panel). Moreover, the growing cells could form colonies on agar plates of RMGCp but not RMGCpSp (lower panel). Speculatively, after counterselection the cells have lost the clmPheS-expressing plasmid pSsp-RFP, thus becoming resistant to 4-CP and sensitive to spectinomycin. Signal of red fluorescence in the transformants before counterselection could be detected, whereas not detectable in those undergone counterselection (Fig. 2D), further confirming the loss of pSsp-RFP after counterselection. All these combined results suggested that a clmPheS-expressing plasmid can be completely discarded by the cells immediately after culturing in an RMGCp medium, demonstrating the sufficiency of a single round of 4-CP counterselection for efficient plasmid curing.

### clmPheS allows for rapid multi-round CRISPR-Cas-based genome editing

We have previously made gene knockouts using the endogenous Type I-F CRISPR-Cas-based genome editing technology in *Z. mobilis* DRM1, a genetic host derived from ZM4 [13]. Despite of the high efficacy of the technology per se, the overall efficiency of multi-round genomic modifications was badly influenced by the difficulty in curing the stable pL2R scaffold-based genome editing plasmids. Since we have constructed the clmPheS counterselection marker, we would like to make use of it to facilitate plasmid curing, expediting multi-round genome editing.

**Fig. 3:**
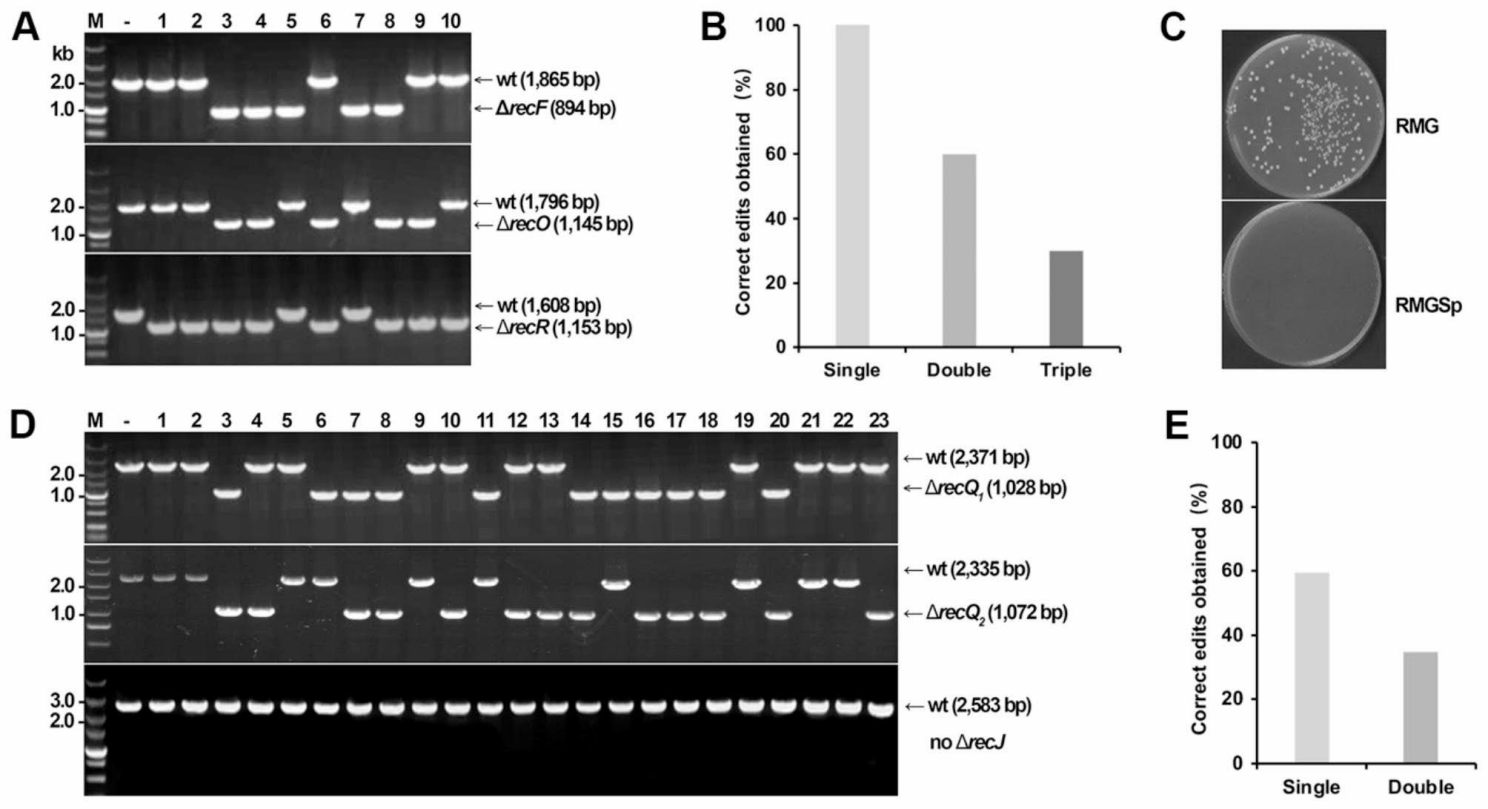
Multi-round multiplex genome editing with the assistance of clmPheS. (**A**) Colony PCR screening of deletion mutants yielded in the first round of multiplex genome editing simultaneously targeting the *recFOR* genes. Predicted sizes of PCR products were indicated. -, PCR amplification using chromosomal DNA of *Z. mobilis* ZM4 as a DNA template; M, DNA size marker. (**B**) Percentages of the correct colonies of single, double, and triple deletions verified in (**A**). (**C**) Examination of the counterselection effect of 4-CP on curing of the genome editing plasmid. (**D**) Colony PCR screening of deletion mutants yielded in the second round of multiplex genome editing simultaneously targeting the *recJQ*_*1*_*Q*_*2*_ genes. Predicted sizes of PCR products were indicated. -, PCR amplification using chromosomal DNA of *Z. mobilis* ZM4 as a DNA template; M, DNA size marker. (**E**) Percentages of the correct colonies of single and double deletions verified in (**D**)

Following the protocol we previously established [13, 14], we constructed multiplex genome editing plasmids, pCE-*recFOR* and pCE-*recJQ*_*1*_*Q*_*2*_, for simultaneously deleting the corresponding *recFOR* (*ZMO1584*, *ZMO0673*, and *ZMO0812*, respectively) and *recJQ*_*1*_*Q*_*2*_ (*ZMO1231*, *ZMO1214*, and *ZM1417*, respectively) genes that are involved in the RecF repair pathway [43]. In addition to the essential elements, i.e. an artificial CRISPR array and the donor DNA fragments, the clmPheS expression cassette was also put on each of the plasmids. Electroporating pCE-*recFOR* into the *Z. mobilis* DRM1 cells yielded more than 200 transformants, among which 10 were randomly picked up and genotypically characterized by colony PCR and Sanger sequencing. Results of colony PCR amplifying the fragments encompassing the targeted regions showed that 5 colonies had *recF* deletion, 5 colonies harbored the mutated *recO* alleles, and 8 colonies contained deletion of *recR* in their chromosomes, respectively giving PCR products with predicted sizes of 894 bp in Δ*recF*, 1,145 bp in Δ*recO*, and 1,153 bp in Δ*recR* strains (Fig. 3A). Collectively, all the tested cells harbored at least one of the expected deletions, showing an overall 100% editing efficiency. The rates of obtaining correct double (Δ*recFO*, Δ*recFR*, and Δ*recOR*) and triple (Δ*recFOR*) deletion mutants were 60% (6/10) and 30% (3/10), respectively (Fig. 3B).

To allow for a second round of genome editing with pCE-*recJQ*_*1*_*Q*_*2*_ in a Δ*recFOR* strain, we grew up the cells in an RMGCp liquid medium for 24-hour incubation to let them get rid of the pCE-*recFOR* plasmid. For double check, we made a 10^5^-fold dilution of the cells and spread them onto an RMG agar plate with or without spectinomycin. As shown in Fig. 3C, the cells could no longer grow on the spectinomycin-containing plate, indicating successful and high-efficiency curing of the editing plasmids. We next prepared competent cells of Δ*recFOR* for hosting a second round of transformation with the pCE-*recJQ*_*1*_*Q*_*2*_ plasmid. To our surprise, totally only 23 transformants were obtained from the pCE-*recJQ*_*1*_*Q*_*2*_ transformation, giving a much lower transformation efficiency than that seen in our previous studies. Looking for an explanation, we grew up all the 23 transformants and performed colony PCR and Sanger sequencing genotypic characterizations. The results showed that 16 colonies possessed deletion of at least one of the two *recQ* genes (Fig. 3D), which included 8 double deletants, exhibiting actually considerably high editing efficiencies (59.56% and 34.78% for single and double deletions, respectively) (Fig. 3E). However, all the *recJ* alleles remained a wild type, suggesting the essentiality of this gene for cell viability and possibly accounting for the observed low transformation efficiency. The mutations have been confirmed by Sanger sequencing of the PCR products (Fig. S1).

Conclusively, clmPheS has endowed very fast removal of genome editing plasmids from *Z. mobilis* cells. With its assistance, we have attained two rounds of CRISPR-Cas-based genome editing in 5 days where a total of 5 genes have been removed from the chromosome, which is obviously advanced because previously we had to spend at least two weeks to attain so [13].

### clmPheS enables scarless genetic manipulation of native plasmids

**Fig. 4:**
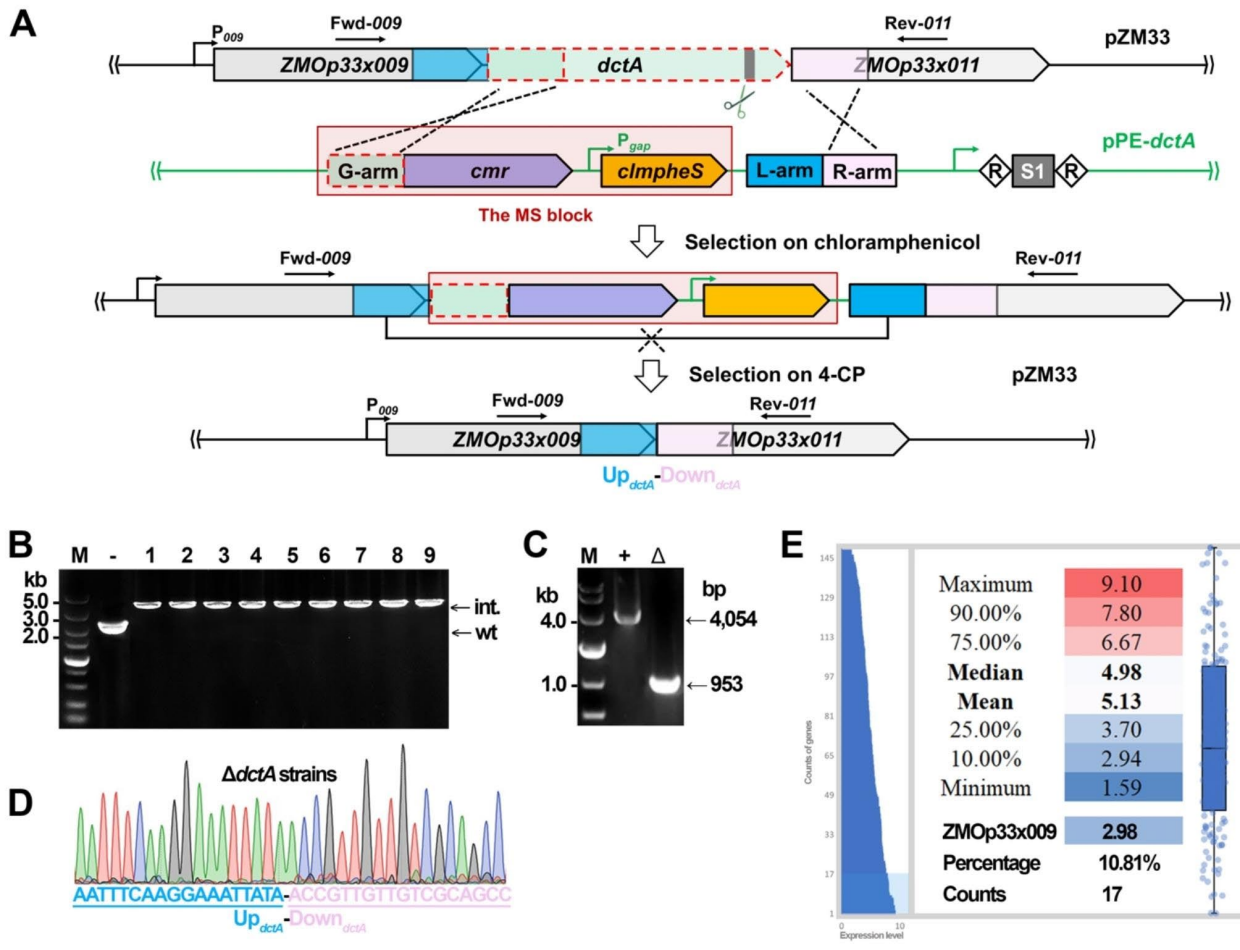
Native pZM33 plasmid editing with the assistance of clmPheS. (**A**) Schematic showing design of the deletion of the *dctA* gene located on the pZM33 native plasmid of *Z. mobilis* ZM4. The pPE-*dctA* plasmid harbors a MS block consisting of the chloramphenicol resistance gene, an artificial CRISPR with a spacer targeting a sequence in the *dctA* gene, and two arms (L-arm and R-arm) for homologous recombination. While transformants with the integration of the MS block into pZM33 are selected on chloramphenicol; the expected deletant is selected on 4-CP upon recombination between two L-arms, resulting in a sequence junction of the L- and R-arms. P_*009*_, promoter of the *ZMOp33x009* gene; *cmr*, chloramphenicol resistance gene. (**B**) PCR screening of *dctA*::MS recombinants using the primer set of Fwd-*009* and Rev-*011* indicated in (**A**). PCR products amplified from the transformants carrying the pZM33 plasmid with or without the integration of the MS block are indicated as int. and wt, respectively. -, PCR amplification using the total DNA of *Z. mobilis* ZM4 as a DNA template. M, DNA size marker. (**C**) PCR amplification verifying the Δ*dctA* mutant. The predicted sizes of PCR products in the *dctA*::MS recombinant (+) and Δ*dctA* mutant (D) are indicated with arrows. M, DNA size marker. (**D**) Representative chromatograph of Sanger sequencing result. The junction of the sequences immediately upstream and downstream of the *dctA* gene corresponding to the L- and R-arms pictured in (**A**) is showcased. (**E**) Statistical analysis of expression levels of the native plasmids-borne genes based on 3 RNA-Seq transcriptomic data. Gene expression levels and the corresponding gene counts are indicated. The expression value of *ZMOp33x009* and the percentage and counts of genes with expression levels below this value are shown at the bottom

Next, to challenge scarless modifications of a nonessential native plasmid of *Z. mobilis* ZM4 [27] rather than lose it entirely, a positive and counterselection cassette was carefully designed and included in the pPE-*dctA* plasmid for knocking out the native pZM33 plasmid-borne non-essential *dctA* (*ZMOp33x010*) gene coding for a C4-dicarboxylate transporter [36]. Briefly, as shown in Fig. 4A, a fragment consisting of the first 327 bp coding sequences of the *dctA* gene was presented as the gene arm (G-arm) and 5’-terminally fused to the chloramphenicol resistance gene (*cmr*), generating a fusion marker for positive selection. This fusion, together with a clmPheS expression cassette, formed a marker-clmPheS (MS) block. Two 300-bp sequneces immediately upstream and downstream, respectively, of the *dctA* gene were set as the left and the right arms (L-arm and R-arm) and put behind the MS block. We inferred that after electroporating the pPE-*dctA* plasmid into the host cells, the fusion marker could not be expressed from this episomal plasmid due to missing a promoter; however, if recombination between the G-arm and the R-arm occurred, the MS block would replace the *dctA* gene, making the fusion marker be coexpressed with the *ZMOp33x009* gene by the upstream promoter P_*009*_ to enable selection for chloramphenicol-resistant transformants.

Electraporation of the pPE*-dctA* plasmid into the *Z. mobilis* ZM4 cells yielded a total of 9 transformants on the RMGSpCm plates, suggesting that CRISPR-targeting had led to elimination of the native pZM33 plasmid from most of the cells. Successful integration of the MS block might have happened to confer these obtained transfromants chloramphenicol resistance. For verification, we performed colony PCR analysis of the transformants using a primer pair of Fwd-*009* + Rev-*011* (Fig. 4A). PCR products with a predicted size of 2,249 or 4,054 bp were expected to be amplified from the wild-type allele (wt) or the designed mutant allele with the integration (int.), respectively. The results showed that a band of ca. 4 kb was amplified in all the 9 strains, suggesting that they all harbored the integration of the MS block (Fig. 4B); and the integration could be efficiently excised via recombination between the homologous L-arms by 0.2 mM 4-CP selection, which was subsequently confirmed by PCR analysis using the same primer set amplifying products with a predicted size of 953-bp and Sanger sequencing (Fig. 4C & D). Interestingly, by taking the same strategy, a fragment covering genes coding for a Type I restriction-modification system (*ZMOp32x025*/*hsdR*_*p*_, *ZMOp32x026*/*hsdM*_*p*_ and *ZMOp32x029*/*hsdS*_*p*_) [6] was also successfully removed from the native pZM32 plasmid, albeit less efficiently (Fig. S2).

Despite the successes, an outstanding concern has to be addressed, that is, since in each case the native promoter of a target gene was expected to drive the expression of the fusion marker to render the cells chloramphenicol resistance, possibly only genes with relatively stronger promoters can be edited. To this end, we performed transcriptomic analysis to reveal the expression of the native plasmids-carried genes. We found that the expression level of *ZMOp33x009* is significantly lower than the mean and median values of all genes. Significantly, only a small portion (10.81%) of the predicted ORFs (in a total of 150) [36], i.e. 17 genes, show lower expression levels than that of *ZMOp33x009* (Fig. 4E & Table S4). We noticed that, of the 17 genes, 11 are located at the distal end of different operons, explaining their relatively lower expression levels (Table S4). Moreover, we could not rule out the possibility that, even if the expression level of the *cmr* gene were lower than that of *ZMOp33x009*, it may still sufficient for conferring on cells chloramphenicol resistance. All these combined results would suggest the universal functionality of this system for native plasmids editing in *Z. mobilis* ZM4.

We observed that very few transformants could be yielded, being 9 and 1 in the transformations with pPE*-dctA* and pPE*-hsd*, respectively (Fig. 4 & S2), reflecting that CRISPR-targeting has made most of the cells lose the targeted plasmids and hence perturbing the integration of the fusion marker to code for the chloramphenicol resistance. This is in line with the demonstration that due to the non-essentiality of the native plasmids, they can be easily removed as a whole by CRISPR-targeting [15]. It might be suggestive of the difficulty in modifying a given gene rather than lose the entire native plasmid carrying it. Nonetheless, we have already achieved modification of the native plasmids, which allows us to investigate functions of the plasmid genes as well as the functions and contributions of these plasmids to cell fitness in the future. When stable inheritance of metabolic operons in commercial strains is in need, the native pZM33 plasmid represents a preferred option over the chromosome, as it can be stably maintained in the cells and its relatively higher copy number [36] would favor elevated production levels.

## Conclusions

In this work, we reported the establishment of one of the most convenient counterselection markers available for *Z. mobilis* based on a conditional lethal mutant of PheS, clmPheS. It can be simply expressed from an episomal plasmid without the requirement for silencing the original *pheS* gene or other pretreatment of the host cells. Coupling it with the endogenous Type I-F CRISPR-based genome engineering platform, rapid multi-round multiplex engineering of the chromosome has been efficiently accomplished, allowing deletion of 5 genes in only 5 days. It would be of particular importance in functional characterization of pathways or systems where multiple genes are typically involved. In addition, it has allowed the development of a method for scarless modification of native plasmids in *Z. mobilis*, thus providing us with a useful tool to demonstrate the functions and contributions of the native plasmids to cell fitness. The method developed here would be also useful for other non-model organisms.

### Electronic supplementary material

Below is the link to the electronic supplementary material.

Supplementary Material 1

## Data Availability

The authors declare that the main data supporting the findings of this work are available within the article and its supplementary information files or from the corresponding authors upon reasonable request.

## References

[1] He MX, Wu B, Qin H, Ruan ZY, Tan FR, Wang JL, Shui ZX, Dai LC, Zhu QL, Pan K (2014). *Zymomonas mobilis*: a novel platform for future biorefineries. Biotechnol Biofuels.

[2] Yang S, Fei Q, Zhang Y, Contreras LM, Utturkar SM, Brown SD, Himmel ME, Zhang M (2016). *Zymomonas mobilis* as a model system for production of biofuels and biochemicals. Microb Biotechnol.

[3] Mohagheghi A, Evans K, Chou YC, Zhang M (2002). Cofermentation of glucose, xylose, and arabinose by genomic DNA-integrated xylose/arabinose fermenting strain of *Zymomonas mobilis* AX101. Appl Biochem Biotechnol.

[4] Wang X, He Q, Yang Y, Wang J, Haning K, Hu Y, Wu B, He M, Zhang Y, Bao J (2018). Advances and prospects in metabolic engineering of *Zymomonas mobilis*. Metab Eng.

[5] Strazdina I, Balodite E, Lasa Z, Rutkis R, Galinina N, Kalnenieks U (2018). Aerobic catabolism and respiratory lactate bypass in *ndh*-negative *Zymomonas mobilis*. Metab Eng Commun.

[6] Lal PB, Wells F, Myers KS, Banerjee R, Guss AM, Kiley PJ (2021). Improving mobilization of foreign DNA into *Zymomonas mobilis* strain ZM4 by removal of multiple restriction systems. Appl Environ Microbiol.

[7] Geng B, Huang X, Wu Y, He Q, Yang S. Identification and characterization of genes related to ampicillin antibiotic resistance in *Zymomonas mobilis*. Antibiot (Basel). 2022;11:1476. 10.3390/antibiotics11111476 PMC9686808 36358131

[8] Bochner B, Gomez V, Ziman M, Yang S, Brown SD (2010). Phenotype microarray profiling of *Zymomonas mobilis* ZM4. Appl Biochem Biotechnol.

[9] Lal PB, Wells FM, Lyu Y, Ghosh IN, Landick R, Kiley PJ (2019). A markerless method for genome engineering in *Zymomonas mobilis* ZM4. Front Microbiol.

[10] Lal PB, Wells F, Kiley PJ (2022). Creation of markerless genome modifications in a nonmodel bacterium by fluorescence-aided recombineering. Methods Mol Biol.

[11] Zou SL, Hong LF, Wang C, Jing X, Zhang MH (2012). Construction of an unmarked *Zymomonas mobilis* mutant using a site-specific FLP recombinase. Food Technol Biotech.

[12] Schweizer HP (2003). Applications of the *Saccharomyces cerevisiae* FLP-FRT system in bacterial genetics. J Mol Microbiol Biotechnol.

[13] Zheng Y, Han J, Wang B, Hu X, Li R, Shen W, Ma X, Ma L, Yi L, Yang S, Peng W (2019). Characterization and repurposing of the endogenous type I-F CRISPR-Cas system of *Zymomonas mobilis* for genome engineering. Nucleic Acids Res.

[14] Hao Y, Wang Q, Li J, Yang S, Zheng Y, Peng W. Double nicking by RNA-directed Cascade-nCas3 for high-efficiency large-scale genome engineering. Open Biol 2022; 12. 10.1098/rsob.210241 PMC8753164 35016549

[15] Geng B, Liu S, Chen Y, Wu Y, Wang Y, Zhou X, Li H, Li M, Yang S (2022). A plasmid-free *Zymomonas mobilis* mutant strain reducing reactive oxygen species for efficient bioethanol production using industrial effluent of xylose mother liquor. Front Bioeng Biotechnol.

[16] Xia J, Liu CG, Zhao XQ, Xiao Y, Xia XX, Bai FW (2018). Contribution of cellulose synthesis, formation of fibrils and their entanglement to the self-flocculation of *Zymomonas mobilis*. Biotechnol Bioeng.

[17] Senthilkumar V, Rajendhran J, Busby SJ, Gunasekaran P (2009). Characterization of multiple promoters and transcript stability in the *sacB-sacC* gene cluster in *Zymomonas mobilis*. Arch Microbiol.

[18] Braga A, Gomes D, Rainha J, Cardoso BB, Amorim C, Silverio SC, Fernandez-Lobato M, Rodrigues JL, Rodrigues LR (2022). Tailoring fructooligosaccharides composition with engineered *Zymomonas mobilis* ZM4. Appl Microbiol Biotechnol.

[19] Wu S, Xu R, Su M, Gao C, Liu Y, Chen Y, Luan G, Jia X, Wang R (2022). A *pyrF*-based efficient genetic manipulation platform in *Acinetobacter baumannii* to explore the vital DNA components of adaptive immunity for I-F CRISPR-Cas. Microbiol Spectr.

[20] Jones RA, Yee WX, Mader K, Tang CM, Cehovin A. Markerless gene editing in *Neisseria gonorrhoeae*. Microbiology. 2022;168(6):001201. 10.1099/mic.0.001201 35763318

[21] Chen L, Liu H, Wang L, Tan X, Yang S (2021). Synthetic counter-selection markers and their application in genetic modification of *Synechococcus elongatus* UTEX2973. Appl Microbiol Biotechnol.

[22] Bibek GC, Zhou P, Wu C (2023). HicA toxin-based counterselection marker for allelic exchange mutations in *Fusobacterium nucleatum*. Appl Environ Microbiol.

[23] Ishikawa M, Yokoe S, Kato S, Hori K (2018). Efficient counterselection for *Methylococcus capsulatus* (bath) by using a mutated *pheS* gene. Appl Environ Microbiol.

[24] Xin Y, Guo T, Mu Y, Kong J (2017). Development of a counterselectable seamless mutagenesis system in lactic acid bacteria. Microb Cell Fact.

[25] Liu Y, He X, Zhu P, Cheng M, Hong Q, Yan X (2020). *pheS (AG)* based rapid and efficient markerless mutagenesis in *Methylotuvimicrobium*. Front Microbiol.

[26] Carr JF, Danziger ME, Huang AL, Dahlberg AE, Gregory ST (2015). Engineering the genome of *Thermus thermophilus* using a counterselectable marker. J Bacteriol.

[27] Wang Y, Yuan L, Tao H, Jiang W, Liu C (2018). *pheS** as a counter-selectable marker for marker-free genetic manipulations in *Bacillus anthracis*. J Microbiol Methods.

[28] Schuster CF, Howard SA, Grundling A (2019). Use of the counter selectable marker PheS* for genome engineering in *Staphylococcus aureus*. Microbiol (Reading).

[29] Gao G, Wei D, Li G, Chen P, Wu L, Liu S, Zhang Y (2022). Highly effective markerless genetic manipulation of *Streptococcus suis* using a mutated PheS-based counterselectable marker. Front Microbiol.

[30] Kast P, Hennecke H (1991). Amino acid substrate specificity of *Escherichia coli* phenylalanyl-tRNA synthetase altered by distinct mutations. J Mol Biol.

[31] Miyazaki K (2015). Molecular engineering of a PheS counterselection marker for improved operating efficiency in *Escherichia coli*. Biotechniques.

[32] Yang S, Mohagheghi A, Franden MA, Chou YC, Chen X, Dowe N, Himmel ME, Zhang M (2016). Metabolic engineering of *Zymomonas mobilis* for 2,3-butanediol production from lignocellulosic biomass sugars. Biotechnol Biofuels.

[33] Okamoto T, Nakamura K (1992). Simple and highly efficient transformation method for *Zymomonas mobilis* - electroporation. Biosci Biotechnol Biochem.

[34] Horton RM, Cai ZL, Ho SN, Pease LR (1990). Gene splicing by overlap extension: tailor-made genes using the polymerase chain reaction. Biotechniques.

[35] Xia Y, Li K, Li J, Wang T, Gu L, Xun L (2019). T5 exonuclease-dependent assembly offers a low-cost method for efficient cloning and site-directed mutagenesis. Nucleic Acids Res.

[36] Yang S, Vera JM, Grass J, Savvakis G, Moskvin OV, Yang Y, McIlwain SJ, Lyu Y, Zinonos I, Hebert AS (2018). Complete genome sequence and the expression pattern of plasmids of the model ethanologen *Zymomonas mobilis* ZM4 and its xylose-utilizing derivatives 8b and 2032. Biotechnol Biofuels.

[37] Chudakov DM, Matz MV, Lukyanov S, Lukyanov KA (2010). Fluorescent proteins and their applications in imaging living cells and tissues. Physiol Rev.

[38] Utturkar S, Dassanayake A, Nagaraju S, Brown SD (2020). Bacterial differential expression analysis methods. Methods Mol Biol.

[39] Rice P, Longden I, Bleasby A (2000). EMBOSS: the European Molecular Biology Open Software Suite. Trends Genet.

[40] Kim D, Paggi JM, Park C, Bennett C, Salzberg SL (2019). Graph-based genome alignment and genotyping with HISAT2 and HISAT-genotype. Nat Biotechnol.

[41] Liao Y, Smyth GK, Shi W (2014). featureCounts: an efficient general purpose program for assigning sequence reads to genomic features. Bioinformatics.

[42] Song H, Yang Y, Li H, Du J, Hu Z, Chen Y, Yang N, Mei M, Xiong Z, Tang K (2022). Determination of nucleotide sequences within promoter regions affecting promoter compatibility between *Zymomonas mobilis* and *Escherichia coli*. ACS Synth Biol.

[43] Pages V (2016). Single-strand gap repair involves both RecF and RecBCD pathways. Curr Genet.

