# Supplementary material for: Development of a counterselectable system for rapid and efficient CRISPR-based genome engineering in Zymomonas mobilis
Source: Microb Cell Fact. 2023 Oct 13;22:208. doi: 10.1186/s12934-023-02217-9 (PMC10571335; doi:10.1186/s12934-023-02217-9)
Supplement: Supplementary file 1 — Supplementary Material 1 [file 12934_2023_2217_MOESM1_ESM.doc]

**Fig. S1** Representative chromatographs of Sanger sequencing results of *rec* gene deletion mutants shown in Figure 3. The up-flanking (UF) and down-flanking (DF) sequences of each target gene are underlined in blue and red, respectively.

**
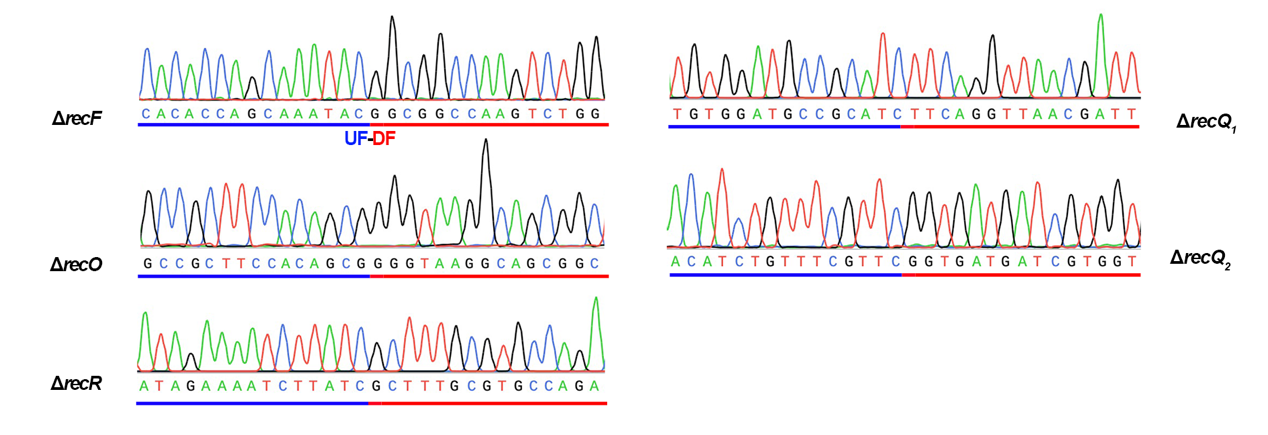
**

**Fig. S2 Native pZM32 plasmid editing with the assistance of clmPheS.** (**A**) Schematic showing design of the deletion of the *hsd* operon located on the pZM32 native plasmid of *Z. mobilis* ZM4. The pPE-*hsd* plasmid harbors a MS block consisting of chloramphenicol resistance gene, an artificial CRISPR with a spacer targeting a sequence in the *hsdR_p_* gene, and two arms (L-arm and R-arm) for homologous recombination. While transformants with the integration of the MS block into pZM32 are selected on chloramphenicol; the expected deletant is selected on 4-CP upon recombination between two L-arms, resulting in a sequence junction of the L- and R-arms. P*_hsd_*, promoter of the *hsd* operon; *cmr*, chloramphenicol resistance gene. (**B**) PCR screening of *hsd*::MS recombinants using the primer set of Fwd-*hsd* and Rev-*hsd* indicated in (**A**). PCR products amplified from the transformants carrying the pZM32 plasmid with or without the integration of the MS block are indicated as int. and wt, respectively. M, DNA size marker. (**C**) PCR amplification verifying the Δ*hsd* mutant. The predicted sizes of PCR products in the *hsd*::MS recombinant (+) and Δ*hsd* mutant (Δ) are indicated with arrows. M, DNA size marker. (**D**) Representative chromatograph of Sanger sequencing result. The junction of the sequences immediately upstream and downstream of the *hsd* operon corresponding to the L- and R-arms pictured in (**A**) is showcased.

**
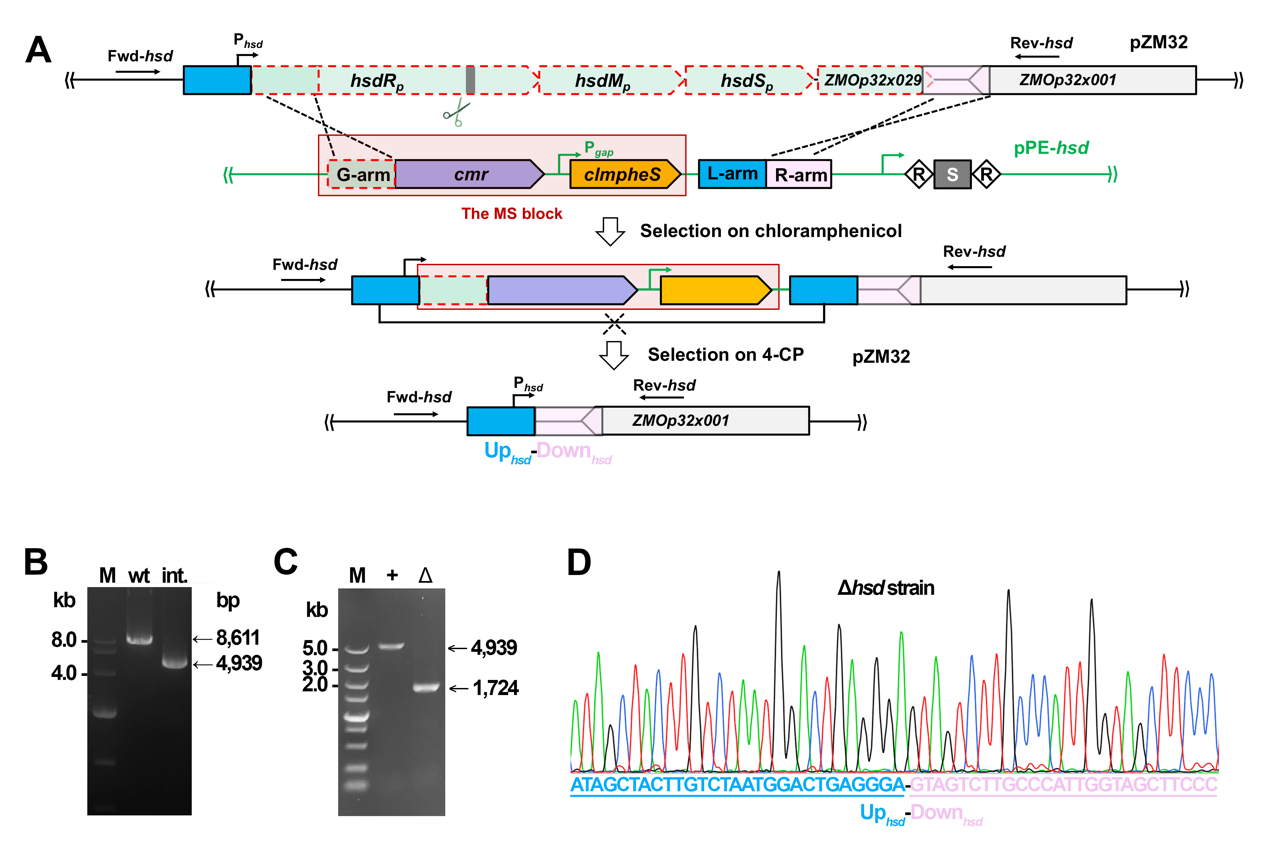
**

**Table S1. *Zymomonas mobilis* strains used or constructed in this work.**

| **Strains** | **Genotype and features** | **Source or reference** |
| --- | --- | --- |
| ZM4 | A wild-type strain *Z. mobilis* subsp. *mobilis ZM4* | Seo *et al*. 2005 |
| DRM1 | ZM4 derivative with the entire *ZMO0028* gene deleted | Zheng *et al.* 2019 |
| ∆*recF* | DRM1 derivative with the entire *recF* gene deleted | This work |
| ∆*recO* | DRM1 derivative with the entire *recO* gene deleted | This work |
| ∆*recR* | DRM1 derivative with the entire *recR* gene deleted | This work |
| ∆*recQ_1_* | DRM1 derivative with the entire ∆*recQ_1_* gene deleted | This work |
| ∆*recQ_2_* | DRM1 derivative with the entire ∆*recQ_2_* gene deleted | This work |
| ∆*recFR* | DRM1 derivative with the genes of *recF* and *recR* deleted | This work |
| ∆*recOR* | DRM1 derivative with the genes of *recO* and *recR* deleted | This work |
| ∆*recFOR* | DRM1 derivative with the genes of recF, *recO* and *recR* deleted | This work |
| ∆*recQ_1_Q_2_* | DRM1 derivative with the genes of *recQ_1_* and *recQ_2_* deleted | This work |
| ∆*dctA*::MS | DRM1 derivative with the replacement of the *dctA* gene of the native pZM33 plasmid with the chloramphenicol resistance marker-PheS (MS) block | This work |
| ∆*hsd*::MS | DRM1 derivative with the replacement of the 3-gene *hsd* operon located on the native pZM32 plasmid with the chloramphenicol resistance marker-PheS (MS) block | This work |

**Table S2. Plasmids used or constructed in this work.**

| **Plasmids** | **Genotype and features** | **Source or reference** |
| --- | --- | --- |
| pEZ15Asp | A *Z. mobilis-E. coli* shuttle vector contains a *Z. mobilis* origin and the *E. coli* origin 15A; Sp^r^ | Yang *et al.* 2016 |
| pSsp | pEZ15Asp carrying a clm-PheS expression cassette | This work |
| pSsp-RFP | pSsp carrying an mCherry expression cassette | This work |
| pL2R | pEZ15Asp containing a DNA fragment of two tandem copies of CRISPR repeat; for artificial CRISPR loci construction | Zheng *et al.* 2019 |
| pCE-*recFOR* | pL2R derivative containing a CRISPR locus with three spacers respectively matching protospacers within the genes of *recF*, *recO*, and *recR*, and three ca. 600-bp concatenated donors of recombination arms homologous to the sequences flanking each target | This work |
| pCE-*recJQ_1_Q_2_* | pL2R derivative containing a CRISPR locus with three spacers respectively matching protospacers within the genes of *recJ*, *recQ_1_*, and *recQ_2_*, and three ca. 600-bp concatenated donors of recombination arms homologous to the sequences flanking each target | This work |
| pPE-*dctA* | pL2R derivative containing a CRISPR locus with a spacer matching a protospacer in *dctA*, a donor of recombination arms consisting of a MS block and sequences flanking *dctA* | This work |
| pPE-*hsd* | pL2R derivative containing a CRISPR locus with a spacer matching a protospacer in *hsdR*, a donor of recombination arms consisting of a MS block and sequences flanking the four-gene *hsd* operon | This work |

**Table S3**. Oligonucleotides used in this work. Restriction sites or protruding nucleotides underlined; mutations in bold; sequences overlapped with plasmid shown as italics.

| **Oligonucleotide** | **Sequence (**5’-3’**)** |
| --- | --- |
| Pgap-Fwd | CTAGAGTGATATCAGATCTC*GAGCTC*CGCGGCCGCGTTCGATCAA |
| Pgap-PheS-Rev | TCGGTCATGCTTTCCATCATGTTTATTCTCCTAACTTATT |
| PheS-Pgap-Fwd | AATAAGTTAGGAGAATAAACATGATGGAAAGCATGACCGA |
| PheS(T267A)-Rev | AGAAGGTTC**AGC**GAAGGGGAAATAACTGGGAC |
| PheS(T267A)-Fwd | TTCCCCTTC**GCT**GAACCTTCTATGGAAGTTGA |
| PheS(A322G)-Rev | GCAACCAAA**GCC**AAAACCTTGCCAGACATTGG |
| PheS(A322G)-Fwd | CAAGGTTTT**GGC**TTTGGTTGCGGTATCGACCG |
| PheS-Rev | GGGTCAAATAAAGGGTCAAA*CCCGGG*TCATGCGCCGACTCCTCCT |
| Ptet-mC(PheS)-Fwd | CCAGCTCACCGTCTGAATTCAAGACGGATGTGATCATTTT |
| Ptet-mC(PheS)-Rev | ACGCGGCCGCGGAGCTCGAGCTAGCAATATTCCTTCCGGA |
| pEZ15A(mc)-Fwd | TCCGGAAGGAATATTGCTAGCTCGAGCTCCGCGGCCGCGT |
| pEZ15A(mc)-Rev | AAAATGATCACATCCGTCTTGAATTCAGACGGTGAGCTGG |
| RecF-gR-Fwd | GAAAGACATTACAGCCGTTATGAAGCAGCGATGCGA |
| RecF-gR-Rev | GAACTCGCATCGCTGCTTCATAACGGCTGTAATGTC |
| RecF-up-Fwd | AGGTCACCAGCTCACCGTCT*GAATTC*CGCTGCAAGTTAGGCAAGC |
| RecF-up-Rev | GTCATCCAGACTTGGCCGCCGTATTTGCTGGTGTGACCGG |
| RecF-down-Fwd | CCGGTCACACCAGCAAATACGGCGGCCAAGTCTGGATGAC |
| RecF-down-Rev | CTCGAGAGATCTGATATCAC*TCTAGA*CTTCGAACACCATATGGTGC |
| RecO-gR-Fwd | GAAAGTTGCATCATAACCGGACAAGAAGCCTGTCTG |
| RecO-gR-Rev | GAACCAGACAGGCTTCTTGTCCGGTTATGATGCAAC |
| RecO-up-Fwd | AGGTCACCAGCTCACCGTCT*GAATTC*GATCGTAATGCGGGACGGG |
| RecO-up-Rev | AATCAGCCGCTGCCTTACCCCGCTGTGGAAGCGGCTCGTT |
| RecO-down-Fwd | AACGAGCCGCTTCCACAGCGGGGTAAGGCAGCGGCTGATT |
| RecO-down-Rev | CTCGAGAGATCTGATATCAC*TCTAGA*CCAGCGGCCAGAAAGGGGA |
| RecR-gR-Fwd | GAAAGACCCCAAGCGGGATGATGCCTTGCTGTGTGT |
| RecR-gR-Rev | GAACACACACAGCAAGGCATCATCCCGCTTGGGGTC |
| RecR-up-Fwd | AGGTCACCAGCTCACCGTCT*GAATTC*CCGCGACAACCGCAACATC |
| RecR-up-Rev | CTACAGGAAGACCATGCGCCGGGCATAACCGCCAGACTCT |
| RecR-down-Fwd | AGAGTCTGGCGGTTATGCCCGGCGCATGGTCTTCCTGTAG |
| RecR-down-Rev | CTCGAGAGATCTGATATCAC*TCTAGA*GGTCTTCTCACTTCGGCATA |
| RecJ-gR-Fwd | GAAAGGCATTGCCGCTCTATTGGATGTTGCAGGCGT |
| RecJ-gR-Rev | GAACACGCCTGCAACATCCAATAGAGCGGCAATGCC |
| RecJ-up-Fwd | AGGTCACCAGCTCACCGTCT*GAATTC*CGTCATAGCCGCCGACTTC |
| RecJ-up-Rev | ACCCATAGCTTGCGATCACGCCATGCCTGACCCAGAGCAG |
| RecJ-down-Fwd | CTGCTCTGGGTCAGGCATGGCGTGATCGCAAGCTATGGGT |
| RecJ-down-Rev | CTCGAGAGATCTGATATCAC*TCTAGA*GGCGAAGCTCGATTTAATCT |
| RecQ1-gR1-Fwd | GAAAATCCTGATGACTATATCCATCGCATTGGCCGC |
| RecQ1-gR1-Rev | GAACGCGGCCAATGCGATGGATATAGTCATCAGGAT |
| RecQ1-up-Fwd | AGGTCACCAGCTCACCGTCT*GAATTC*GGTGCCGCCGCTGTTTCTG |
| RecQ1-up-Rev | GCAACAGGCTTCACACGCCGGGCCTAGATCGGCAAAGCTC |
| RecQ1-down-Fwd | GAGCTTTGCCGATCTAGGCCCGGCGTGTGAAGCCTGTTGC |
| RecQ1-down-Rev | CTCGAGAGATCTGATATCAC*TCTAGA*CGGGTCGGATACGGTTCAGT |
| RecQ2-gR-Fwd | GAAAGCACGAAGCATGGTGCCGATAGAGTGGTTCGC |
| RecQ2-gR-Rev | GAACGCGAACCACTCTATCGGCACCATGCTTCGTGC |
| RecQ2-up-Fwd | AGGTCACCAGCTCACCGTCT*GAATTC*GGTGAAACCACGCCTTCCA |
| RecQ2-up-Rev | CGATCACCACGATCATCACCGAACGAAACAGATGTTTCCG |
| RecQ2-down-Fwd | CGGAAACATCTGTTTCGTTCGGTGATGATCGTGGTGATCG |
| RecQ2-down-Rev | GATCGAACGCGGCCGCGGAG*CTCGAG*CCGAACGCATCCATCTGA |
| dctA-gR-Fwd | GAAAAAGCAACAGGCACTCATCTTTCATGGACGCAA |
| dctA -gR-Rev | GAACTTGCGTCCATGAAAGATGAGTGCCTGTTGCTT |
| G11-arm-Fwd | GGTCACCAGCTCACCGTCTGAATTCATGCCCTCACTTGCATCTAC |
| G11-arm(CmR-pheS)-Rev | TCCAGTGATTTTTTTCTCCATTCCTCCATGGCGCAAAGATT |
| (G11-arm)CmR-pheS-Fwd | GAATCTTTGCGCCATGGAGGAATGGAGAAAAAAATCACTGGATA |
| CmR-pheS(L11-arm)-Rev | GAAAGCATCTCCTGGGCGCGAGCGGAATTTTCCTCAAAAAGACAA |
| (CmR-pheS)L11-arm-Fwd | TTTTTTGTCTTTTTGAGGAAAATTCCGCTCGCGCCCAGGAGATGCT |
| (R11-arm)L11-arm-Rev | TTTATTACATATAGAAACAGACATTATAATTTCCTTGAAATTAAAGACT |
| CmR(pEZ15A)-Fwd | CGGCTTAATTCAGGAGTTAAACATCATGGAGAAAAAAATCACTGG |
| CmR(pEZ15A)-Rev | GCTTGAACGAATTGTTAGACATTACGCCCCGCCCTGCCACTC |
| PheS(pEZ15A)-Fwd | GAGTGGCAGGGCGGGGCGTAATGTCTAACAATTCGTTCAAGC |
| PheS(pEZ15A)-Rev | CCAGTGATTTTTTTCTCCATGATGTTTAACTCCTGAATTAAGCCG |
| hsd-gR-Fwd | GAAAGCGCCAGAGCTGTTTCGATACGGATAGGTGGT |
| hsd-gR-Rev | GAACACCACCTATCCGTATCGAAACAGCTCTGGCGC |
| Ghsd-arm-Fwd | CGTCTGAATTCGCGGCCGCTTCTAGAATAGTGGATACTTTGAACTT |
| Ghsd-arm(CmR-pheS)-Rev | TCCAGTGATTTTTTTCCTTTCGCGGTGGCACGAAAGCA |
| (Ghsd-arm)CmR-pheS-Fwd | GGTGCTTTCGTGCCACCGCGAAAGGAAAAAAATCACTGGATA |
| CmR-pheS(Lhsd-arm)-Rev | CAAAGGCTGTCTTTGCTCCATCGTCATTTTCCTCAAAAAGACAAAAAAG |
| (CmR-pheS)Lhsd-arm-Fwd | CTTTTTTGTCTTTTTGAGGAAAATGACGATGGAGCAAAGACAGCCTTTG |
| (Rhsd-arm)Lhsd-arm-Rev | CTACCAATGGGCAAGACTACTCCCTCAGTCCATTAGACAAG |
| RecF-Fwd | GGCACGTCTTCACTAGCGGT |
| RecF-Rev | ACCGAGACCGAACCATCGTC |
| RecO-Fwd | GCAGCGAATGGCCGGATCTT |
| RecO-Rev | CGGTAACGAGAATGTGCGCC |
| RecR-Fwd | CCGCGATCCATCTGCATGAT |
| RecR-Rev | CATGCTGAAGGCAGGTTGCC |
| RecJ-Fwd | CGGCGCCATATGATGTTCGC |
| RecJ-Rev | GACATGGAACAGGTGATCCG |
| RecQ-Fwd | CTGAAGCGGTCTGGCATGAG |
| RecQ-Rev | GATTCATCAGCATGTGCGGC |
| recQ2-Fwd | GGGTAGCCGGATTCGGCGTC |
| recQ2-Rev | CCATGGCTGTATCCGACTTG |
| 009-Fwd | GCCGTATAGATCATGGACAT |
| 011-Rev | ATGTCCATGATCTATACGGC |
| hsd-Fwd | GCATTAAAGGTTGGACTTTG |
| hsd-rev | AGGAGACAGGCTAGTTAATA |

**Table S4. Plasmid genes with expression levels lower than that of *ZMOp33x009* (2.98).**

| **Gene Name** | **Expression Level** | **Relative location within an operon** |
| --- | --- | --- |
| *ZMOp32x019* | 2.88 | Orphan |
| *ZMOp32x031* | 2.71 | Orphan |
| *ZMOp36x002* | 2.85 | 7^th^ of an 8-gene operon |
| *ZMOp36x003* | 1.59 | 8^th^ of an 8-gene operon |
| *ZMOp36x005* | 1.98 | 2^nd^ of a 4-gene operon |
| *ZMOp36x006* | 2.73 | 3^nd^ of a 4-gene operon |
| *ZMOp36x007* | 2.49 | 4^th^ of a 4-gene operon |
| *ZMOp36x034* | 2.47 | 2^nd^ of a 4-gene operon |
| *ZMOp36x041* | 1.93 | 2^nd^ of a 2-gene operon |
| *ZMOp36x045* | 2.31 | 3^rd^ of a 5-gene operon |
| *ZMOp36x046* | 2.24 | 4^th^ of a 5-gene operon |
| *ZMOp36x047* | 1.95 | 5^th^ of a 5-gene operon |
| *ZMOp36x050* | 2.40 | Orphan |
| *ZMOp39x003* | 2.48 | Orphan |
| *ZMOp39x017* | 1.59 | 2^nd^ of a 2-gene operon |
| *ZMOp39x019* | 2.55 | Orphan |
| *ZMOp39x024* | 2.68 | 3^nd^ of a 3-gene operon |
